# Can an Online Reading Camp Teach 5-Year-Old Children to Read?

**DOI:** 10.3389/fnhum.2022.793213

**Published:** 2022-03-31

**Authors:** Yael Weiss, Jason D. Yeatman, Suzanne Ender, Liesbeth Gijbels, Hailley Loop, Julia C. Mizrahi, Bo Y. Woo, Patricia K. Kuhl

**Affiliations:** ^1^Institute for Learning & Brain Sciences, University of Washington, Seattle, WA, United States; ^2^Department of Speech and Hearing Sciences, University of Washington, Seattle, WA, United States; ^3^Graduate School of Education, Stanford University, Stanford, CA, United States; ^4^Division of Developmental Behavioral Pediatrics, Stanford University School of Medicine, Stanford, CA, United States; ^5^Department of Psychology, Stanford University, Stanford, CA, United States

**Keywords:** reading acquisition, preschool, online learning, phonological awareness, letter-sound knowledge

## Abstract

Literacy is an essential skill. Learning to read is a requirement for becoming a self-providing human being. However, while spoken language is acquired naturally with exposure to language without explicit instruction, reading and writing need to be taught explicitly. Decades of research have shown that well-structured teaching of phonological awareness, letter knowledge, and letter-to-sound mapping is crucial in building solid foundations for the acquisition of reading. During the COVID-19 pandemic, children worldwide did not have access to consistent and structured teaching and are, as a consequence, predicted to be behind in the development of their reading skills. Subsequent evidence confirms this prediction. With the best evidence-based practice in mind, we developed an online version of a well-structured early literacy training program (Reading Camp) for 5-year-old children. This 2-week online Reading Camp program is designed for pre-K children. It incorporates critical components of the fundamental skills essential to learning to read and is taught online in an interactive, multi-sensory, and peer-learning environment. We measure the participants’ literacy skills and other related skills before and after participating in the online Reading Camp and compare the results to no-treatment controls. Results show that children who participated in the online Reading Camp improved significantly on all parameters in relation to controls. Our results demonstrate that a well-structured evidence-based reading instruction program, even if online and short-term, benefits 5-year-old children in learning to read. With the potential to scale up this online program, the evidence presented here, alongside previous evidence for the efficacy of the in-person program, indicates that the online Reading Camp program is effective and can be used to tackle a variety of questions regarding structural and functional plasticity in the early stages of reading acquisition.

## Introduction

During the COVID-19 pandemic, children worldwide did not have access to consistent and structured teaching. Early on in the pandemic, researchers provided pessimistic predictions regarding the potential effects of the school closure. They predicted significant losses in overall reading and math achievement and increased percentages of students at risk of substantial academic difficulties (Bielinski et al., 2020). Furthermore, predictions indicated that school closure would increase the disparity between students from lower-income and higher-income families (Dron et al., 2020) and between students who showed low as compared to high achievements before the school closure (Kuhfeld et al., 2020).

A recent study confirmed these predictions and showed that second and third graders in over 100 United States school districts nationwide fell about 30 percent behind the expected oral reading fluency scores in fall 2020 and that students at lower-achieving schools fell even farther behind (Domingue et al., 2021). A more recent study included data from 5.5 million students in grades 3–8 and measured their reading and math achievements during the 2020–2021 school year (Lewis et al., 2021). This study demonstrated that students ended the school year 8–12 percentile points lower than historical trends in math and 3–6 percentile points lower in reading achievements. Furthermore, students in high-poverty schools and from racial and ethnic minorities were disproportionately impacted.

Schools in the United States were physically closed in March 2020. Teachers and school systems struggled to provide remote learning for their students. While the school closure during the COVID-19 pandemic had forced teachers to shift to online learning, online learning actually started in the mid-1990s, with at least 2% of United States students (and many more worldwide) participating in some form of K-12 online learning before the pandemic (Black et al., 2021).

Online learning can be synchronous or asynchronous and ranges from digital platforms that teachers use in their classrooms to fully online school. Overall, teaching online requires a unique set of skills and training, including technological, pedagogical, and content knowledge (Moore-Adams et al., 2016). Studies that compared online and face-to-face instructions in pre-pandemic K-12 education have shown that the online programs are as effective as the face-to-face instructions and sometimes are even more effective when measuring the students’ outcomes (Bakia et al., 2012). However, most online schools serve children in 6th–12th grade and are attended primarily by children from white mid-to-high socioeconomic status (SES) who chose this option over the traditional in-person instruction (Molnar et al., 2019; Digital Learning Collaborative, 2020). Furthermore, the level of success of remote learning during the COVID-19 pandemic varied and depended on access to the necessary technology, the quality of remote instructions, the level of student engagement, the academic support at home, and other factors (Bansak and Starr, 2021; Domina et al., 2021).

Literacy is one of the essential skills required to become a successful self-providing human being, and learning to read requires explicit instruction. However, according to the United States Department of Education’s report (National Assessment of Educational Progress [NAEP], 2019), only 35% of elementary students scored proficient on a national assessment of reading skills, and scores did not improve from 2017 to 2019. Based on the recent studies, 2020–2021 school-year results are likely to be even lower. Hence, there is an urgent need to examine the crucial components of early reading acquisition and how it can be taught in an online environment in times of school closure and more generally when online instruction could potentially reach children who cannot attend school.

There is currently limited research on the effectiveness of online reading instruction, especially at the elementary school level. Most of this research has focused on using computer-based programs or blended/hybrid learning (a combination of in-person instructions and digital technology). Previous studies show that computer-based programs benefited kindergarteners and first graders in both phonological awareness (Mitchell and Fox, 2001; Segers and Verhoeven, 2005; Macaruso and Walker, 2008; Wild, 2009; Savage et al., 2013; Prescott et al., 2018) and letter-to-sound knowledge (Segers and Verhoeven, 2005; Macaruso and Walker, 2008; de Graaff et al., 2009; Volpe et al., 2011; Savage et al., 2013), and pre-K children on phonological awareness (Lonigan et al., 2003).

Nevertheless, the unanswered question is: can children learn to read with a fully online teacher-led program? To the best of our knowledge, only one recent study examined a fully online teacher-led synchronous reading intervention program (Beach et al., 2021). This study described the implementation of an online summer reading intervention with low-performing rising second–third graders from low-income families during the COVID-19 pandemic. The summer reading program was based on an in-person program focused on foundational reading skills and adapted to a fully online program. The program took place over 15–22 days. Each day, the children were given one hour of a systematic phonics program (including phonological awareness, letter-word identification, word reading, spelling, sight word reading, and sentence fluency) and one hour of guided reading of leveled texts. Each teacher worked with 1–2 children at a time. The results from this study were encouraging and showed that rising second and third graders improved on the discrete skills that were taught in the program (Beach et al., 2021). While the results of this study looked promising, their main limitations were that they did not include any control group, and they used curriculum-embedded mastery measures instead of externally validated standardized tests. Furthermore, this study targeted a particular group of participants (low-performing, low-income) and thus did not necessarily reflect possible outcomes for a broader range of backgrounds and abilities.

## The Current Study

In the current study, we examine whether 5-year-old pre-K children can learn to read online for the first time. We adapted a proven in-person intervention program, the Language and Literacy Camp (LLC) (Yeatman et al., 2022), into a 2-week fully online program. The in-person LLC included two different intervention programs which were studied in a randomized controlled trial. One program focused on reading skills and the other on oral language skills. In the current study, we test the efficacy of an online adaptation of the Reading Camp. The current study included children with varied levels and backgrounds of pre-reading skills and measured the online Reading Camp’s effectiveness by using standardized and non-standardized tests and comparing the results to no-treatment control participants.

The original goal of the in-person LLC project was to examine how children’s first experiences with reading tune the underlying structure and function of the brain’s visual and language pathways to enable reading (Yeatman et al., 2022). At the beginning of the COVID-19 pandemic and shut down in March 2020, we pivoted to develop the Reading Camp into a fully online program. The original Reading Camp program was developed with the best and most updated evidence-based practice in mind. Based on the National Reading Panel report (National Reading Panel [NRP], 2020), phonological awareness and letter-sound knowledge are the two best predictors of reading acquisition during the first two years in school. Hence, it is essential to include these two components in any reading program for beginners. The NRP report included meta-analyses of studies that examined the effectiveness of phonological awareness and letter-sound knowledge instructions. The results of the phonological awareness meta-analysis indicated that phonological awareness instructions effectively teach children to attend to and manipulate speech sounds, as well as in learning to read and spell, and produce effects that remained strong in both the short and long term. Further, phonological awareness instructions are most effective for preschoolers and kindergarteners and when the instructions are explicit, focus on one or two skills, are taught with letters, taught in small groups (as compared to individually or in a classroom), and last 5–18 h total (National Reading Panel [NRP], 2020). Moreover, the results of the meta-analysis of letter-sound knowledge indicated that systematic letter-sound knowledge instructions are effective in children’s growth in reading, word-reading skills, reading comprehension, spelling, and preventing reading difficulties in children at-risk regardless of the specific method or whether it has been taught individually, in small groups, or a classroom. However, letter-sound knowledge instructions provide the most significant impact on reading growth, word-reading, reading comprehension, and spelling when combined with phonological awareness instructions and when it begins in kindergarten or first grade before children have learned to read independently. Notably, both phonological awareness instructions and letter-sound knowledge instructions were effective across all SES levels.

Based on the results of the NRP meta-analyzes and conclusion (National Reading Panel [NRP], 2020), the online Reading Camp program included the two critical components of early reading instructions: phonological awareness and letter-sound knowledge. The phonological awareness instructions included different teacher-led interactive games that involved various word-sound manipulations. Each phonological awareness session included one game with only one type of manipulation at a time. The phonological awareness activities were organized in a developmentally appropriate order during the online Reading Camp days, from easier to more complex and from larger to smaller phonological units. The letter-sound knowledge instructions included systematic, multi-sensory, and direct instructions on letters’ names, shapes, and corresponding sounds. Once the participants had learned more than five letters, they were also implementing their knowledge in reading short CVC words. Furthermore, additional sessions were dedicated to integrating the participants’ phonological awareness and letter-sound knowledge sessions using short, engaging games and activities. A detailed description of the program and examples is described in the Section “Materials and Methods.”

According to the Simple View of Reading (Hoover and Gough, 1990), reading comprehension is the end goal of reading acquisition and is predicted by listening comprehension and decoding. The decoding aspect includes the two critical components of the Reading Camp programs (i.e., phonological awareness and letter-sound knowledge) and fluency, which is achieved in later phases of reading acquisition once the child becomes automatic in letter-sound mapping. The listening comprehension aspect includes vocabulary, syntax, and background knowledge and is correlated with home environment and book exposure. Studies have shown that reading book to children helps them to build a sense of story and develop vocabulary and comprehension (National Reading Panel [NRP], 2020). Furthermore, children’s home literacy environment and early exposure to literacy activities were found to be highly correlated with emergent reading skills and language development (Strickland, 1989; Bus et al., 1995; Leseman and de Jong, 1998; Sénéchal and LeFevre, 2002; Karrass and Braungart-Rieker, 2005; Weigel et al., 2006; Sénéchal et al., 2008; Sukhram and Hsu, 2012). Despite not being the focus of the current study, we chose to include a short story-time session at the end of each online Reading Camp day to provide some literacy exposure during the camp. At the end of each day, one of the teachers read a children’s book (a different book every day) while showing the scans of the book’s pages on the screen. While this activity was passive listening to a story, the participants were encouraged to make comments and ask questions.

The first goal of the current research was to examine whether pre-K children can learn fundamental reading skills in an online environment and whether their ability to gain from a well-structured online program relates to their SES. The second goal was to set the ground for future examination of the structural and functional changes that occur in the brain when children learn to read, and how this might differ between online and in-person instructions.

## Materials and Methods

### Participants

A total of 670 participants were contacted for this study through The University of Washington (UW) Communications Studies Participants registry with UW Human Subjects Approval that provides subject contact information directly to researchers. Some of these participants had previously participated in studies in our laboratory and agreed to be re-contacted for future research on their consent forms. All experimental procedures were approved by the UW Institutional Review Board, and all participating families gave informed consent^1^ and were compensated monetarily for their time and effort. All families who agreed to have their child participate (a total number of 197 families) completed an initial phone screening interview to determine whether their children met the following criteria: (1) Pre-K child between the age of 5 years and 5 years and 4 months; (2) native English is primary in the home (multi-lingual families were included if English was spoken >65% of the time in the home); and (3) children had no apparent congenital, neurological, or physical abnormalities. Exclusion criteria included: (1) any brain injury and medications that impact cognition; (2) intellectual disability, autism spectrum disorder, mood disorders, and other disorders that impact cognition; and (3) significant and permanent hearing impairments.

After the initial screening process, 188 eligible 5-year-old participants were invited for an online intake session. During the intake session, participants were examined for their uppercase letter knowledge and CVC non-word reading to ensure they did not yet know how to read. After the intake session, 71 participants were excluded from the study because they demonstrated the ability to read, were struggling with the online setting, or were not interested in proceeding with participation. Participants’ families completed an online parental questionnaire that included questions regarding their education, social-economic status, children’s health and development history, language learning history, and family history of dyslexia and reading difficulties. The social-economic status (SES) questions were based on the Hollingshead index (Hollingshead, 1975) and income-to-need ratio (i.e., income divided by the poverty threshold for equivalent family size).

A total number of 117 participants who met all the criteria based on the screening and the intake session were invited to participate as experimental or control group participants. The experimental group included 84 participants who completed the online Reading Camp program described below, but one participant became ill and dropped out, resulting in 83 participants. The control group included 33 participants who underwent the same screening procedures to enroll but did not participate in the online Reading Camp program. The two groups did not differ significantly on SES parameters, including the income-to-need ratio and the average years of parental education. For both groups, the average parental education in years was roughly equivalent to a 4-year college degree, with a wide range extending from elementary to postgraduate level degree completion. For both groups, the range of income-to-need ratio included families at or below the federal poverty line (ratio <1) as well as families ranging well into the upper quadrans of wealth (e.g., ratio = 19.62). The summary statistics of the participants’ gender, age, and SES are presented in Table 1.

**TABLE 1:** Basic demographic information of the experimental and control groups.

		Experimental Group	Control Group	Comparison			
		
Total number of participants		83	33	Test	Value	*df*	sig
**Gender**	Identify as boys	39 (46.98%)	18 (54.54%)	Pearson Chi-Square	1.813	3	0.612
	Identify as girls	42 (50.60%)	13 (39.39%)				
	Other/Prefer not to answer	2 (2.40%)	2 (6.06%)				
**Age at the first session**	Mean age in years	5.09 (0.089)	5.13 (0.071)	*T*-test	–2.107	114	0.037*
**Socio-economic status**	Average years of parental education	17.67 (1.97)	17.51 (2.11)	*T*-test	0.374	114	0.706
	Income-to-need ratio	6.19 (3.57) *N* = 62	7.07 (5.29) *N* = 28	*T*-test	–0.919	88	0.361

*Significance level <0.05.

Participants in both the experimental and control groups went through two online “pre” and two online “post” sessions of standardized and non-standardized tests to measure their progress on metrics related to reading and the effect of the training program on those metrics. For the experimental group, these sessions took place within 2 weeks before and after the online Reading Camp program. For the control group, the “pre” and “post” sessions took place 2–3 weeks apart to match the timeline of the experimental group.

### Procedure

#### Online Reading Camp Program

**FIGURE 1:**
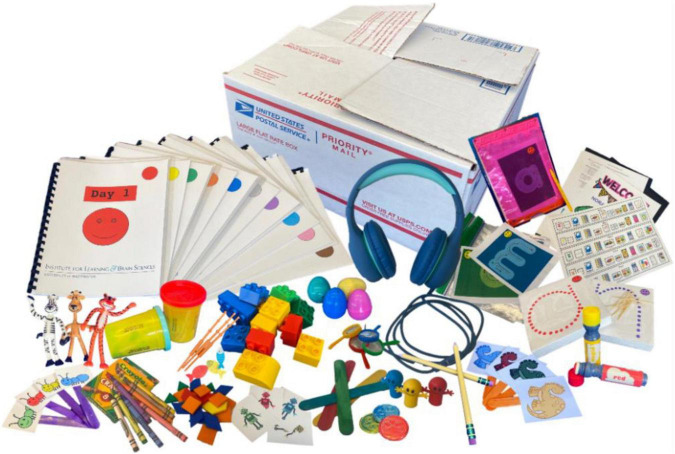
A picture of the supply box sent to participants at home. The box includes child-sized headphones with microphones, binders with relevant worksheets, play dough, stacking building blocks, and other props used during the online Reading Camp.

The Reading Camp program was designed to train preschoolers on fundamental early literacy skills, including phonological awareness, letter-sound knowledge, letter identification, CVC word blending/reading, and exposure to literacy. It is a well-structured training program that incorporates multi-modal learning activities through games, gross-motor and fine-motor movements, and direct instructions and has proven efficacy for in-person instruction (Yeatman et al., 2022). Participants in the experimental group took part in the online Reading Camp program in small groups (14 groups of six participants each) during the period between Fall 2020 – Summer 2021. During this period, the participants experienced varied and constantly changing levels of schooling, including in-person schooling, online schooling, or no schooling at all. The online Reading Camp took place *via* Zoom and lasted 2 weeks, for 5 days a week, with 2.5 h (including breaks) each day. Training sessions were recorded (audio and video) for later documentation of content and analysis of behavior and social interaction. Two teachers taught each group of participants. Activities were primarily conducted in small groups of three children with one teacher (using breakout rooms on Zoom). In addition, each day included activities with the entire group of six participants and two teachers as well. The small groups were mixed and counterbalanced between the camp days so that each child got familiar with all five other children at the same camp. The participants were sent a package via standard mail delivery at home that included all the necessary equipment and materials needed to participate in the program. These included: child-sized headphones with microphones, binders with relevant worksheets, play dough, stacking building blocks, and other props used during the online Reading Camp, as demonstrated in Figure 1. We also provided computer tablets and an internet connection for families who did not have access to these components needed to participate in the online Reading Camp. The online Reading Camp sessions were administered by three teachers with a bachelor’s degree in either Education, Linguistics, or Speech and Hearing Sciences, and with prior experience teaching English to young children in previous lab projects. The teachers of the online Reading Camp program were trained by the developers and teachers of the in-person Reading Camp program (Yeatman et al., 2022) for 2 weeks before administering the program independently.

On each day of the online Reading Camp program, the schedule included two letter-sound knowledge sessions, two phonological awareness sessions, two integration activities sessions, and one story-time session at the end of each day. Each session lasted about 10–20 min with short 5–10 min breaks in between sessions. During the letter-sound knowledge sessions, participants were taught to identify lowercase letters’ names and their corresponding sounds and practiced the letter shapes with gross-motor movements and tracing the letters’ shapes. Children learned two new letters each day, resulting in 20 (out of the 26) letters learned by the end of the program. Each letter-sound knowledge session began with the teacher introducing one letter on the screen along with a picture of an object that starts with the corresponding sound. The teacher encouraged the participants to repeat together the letter name, the word, and its corresponding sound (e.g., “A–apple-/a/” or “P–pig-/p/”). Then, the teacher demonstrated how to create the letter shape with their fingers while providing verbal cues and encouraged the participants to follow. Then, the participants practiced tracing the letter shapes on worksheets. Later, during the first 2 days of the camp, the participants were instructed to identify the target letter within short 2–3-letter words shown on the screen. Starting the third day of the camp, they were instructed to read short CVC words shown on the screen along with a letters’ chart of the letters that they had learned up to that point and their visual cues (objects’ pictures). Finally, each letter-sound knowledge session ended with repeating all the letters learned, while saying together the letter name, the word, its corresponding sound, and demonstrating the letters’ shapes with their fingers.

**FIGURE 2:**
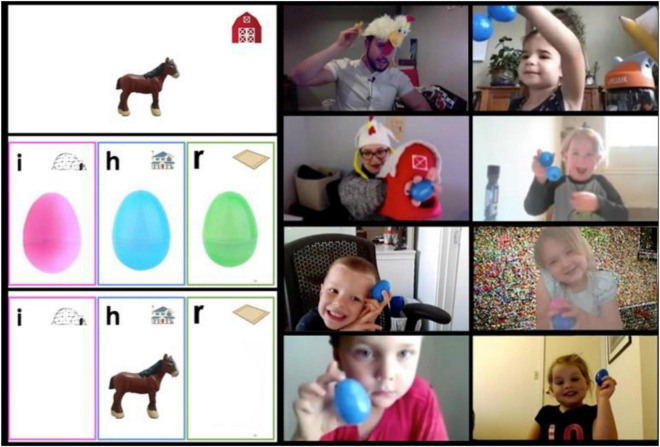
A screenshot demonstrates an example of an integration activity. In this activity the two teachers (upper left) are wearing silly chicken hats to engage the children. With colorful plastic eggs from the supply box, the children pick which egg to hold up to show which letter the item starts with. On the left side, there are three photographs that the children see one after another: first, they see a picture of a horse, next they see a photograph that shows three letter options (“i” for “igloo,” “h” for “house,” and “r” for “rug”), and then finally after voting with their plastic eggs, the horse is revealed to belong to the letter “h.” On the right side, the children are holding up blue eggs because they could tell that the horse starts with the letter “h.”

During the phonological awareness sessions, participants practiced sound manipulations while playing structured games directed by the teacher. These activities were organized according to developmental order of acquisition and introduced by difficulty level, with easier tasks on the initial days of the training program and more difficult ones occurring toward the last days of the training program. The tasks included syllable segmentation and blending, onset-rhyme segmentation and blending, rhyme matching, and CVC words segmentation and blending. During the integration activities sessions, participants practiced letter-sound mapping that incorporates both phonological awareness and letter-sound knowledge, letters identification, letters’ shapes, and reading CVC words through various short, engaging games directed by the teachers. An example of an integration activity is demonstrated in Figure 2.

Finally, at the end of each day, one of the teachers read a short children’s book while the scanned pages were shown on the screen. It was a passive listening activity for the participants. However, participants were encouraged to engage in the story by making comments and asking questions. The daily structure of the program and examples of activities are described in Table 2.

**TABLE 2:** Online reading camp daily schedule with examples.

15 min	Waiting Room	
**10 min**	**Team Building Activities (whole group)**	
**Examples:**	Creating letter shapes with stacking blocks or Playdough	

**30 min**	**Letter-sound knowledge Part 1 (Teacher A)**	**Phonological Awareness Part 1 (Teacher B)**
15 min	Group A	Group B
15 min	Group B	Group A
**Examples:**	Learning the letter“a”- its name, corresponding sound, and relate it to the word “apple”. Repeatedly creating the latter shape with fingers in the air and with a pencil on a worksheet.	Counting syllables by clapping for different words. Blending onset and coda to discover a hidden word. Finding two words that rhyme in a close set. All target words are presented in figures, and not written form to focus on the sounds.

**5 min**	**Short break**	

**18 min**	**Breakout Integration activities Group B (Teacher A)**	**Breakout Integration activities Group A (Teacher B)**
6 min	Center 1	Center 1
6 min	Center 2	Center 2
6 min	Center 3	Center 3
**Examples:**	Taking out “treasures” from a treasure box and sorting by the first letter. “Fishing” words from a pond and sorting by the first letter. Memory game to find a picture and the words’ corresponding letters.	

**10 min**	**Long break**	

**30 min**	**Letter-sound knowledge Part 2 (Teacher A)**	**Phonological Awareness Part 2 (Teacher B)**
15 min	Group A	Group B
15 min	Group B	Group A
**Examples:**	Learning the letter “m” - its name, corresponding sound, and relate it to the word “mouse”. Repeatedly creating the latter shape with fingers in the air and with a pencil on a worksheet.	Blending CVC words (e.g., What word can you find when you hear d-o-g?). Segmenting CVC words (e.g., Break the word “car” into sounds → c-a-r). Building compound words (e.g., What words create the word “mailbox”? →“mail” + “box”). All target words are presented in figures, and not written form to focus on the sounds.

**5 min**	**Short break**	

**18 min**	**Breakout Integration activities Group B (Teacher A)**	**Breakout Integration activities Group A (Teacher B)**
6 min	Center 4	Center 4
6 min	Center 5	Center 5
6 min	Center 6	Center 6
**Examples:**	Helping a hungry puppy find its food by finding the word’s first letter (on the correct bowl). Finding pictures of words that begin with a specified letter. Creating letter shapes with pattern blocks. Reading a CVC word and finding its matching picture.	

**10 min**	**Story Time (Whole group)**	

During the online Reading Camp, parents and other caregivers were instructed to assist their children with logging in to the Zoom sessions and preparing the daily materials to be available for their children when needed. They were instructed to stay within earshot during the time of the online Reading Camp activities in case their children encountered any issues and needed assistance. Furthermore, they were instructed not to provide or prompt answers and to keep siblings away as much as possible. In some cases, when parents’ involvement was beyond the scope of these instructions, the online Reading Camp teachers contacted the family by email or by phone to remind them of the expected level of parental involvement. No specific guidelines were provided to the parents regarding activities after the online Reading Camp sessions. However, they were encouraged to report such activities in a post-camp survey.

#### Standardized and Non-standardized Tests

To examine participants’ progress and compare performance in the experimental and control groups, we measured participants’ reading and related skills using standardized and non-standardized tests before and after the online Reading Camp for the experimental group. For the no-treatment control group, we measured “pre” and “post” at 2–3 week intervals, mirroring the timing of the experimental group’s measures. Specifically, the following tests were administered:

##### Letter Knowledge Test

This test is designed to measure Alphabet knowledge and letter sounds. Participants were shown isolated letters on the screen and instructed to name the letters and their corresponding sounds. This test was administered separately for lowercase and uppercase letters. All 26 letters were presented in random order. For the sounds, we only accepted isolated pronunciation (not adding any vowel) and only short vowels, and hard “G,” “C,” and “X” as correct responses.

##### Woodcock Reading Mastery Tests-Third Edition (WRMT-III)

This standardized test (Woodcock, 2011) is designed to assess reading skills in children and adults. We administered the sub-parts of this test that are suitable for preschoolers. These sub-parts include Phonological Awareness and Rapid Automatic Naming (RAN) of objects and colors. Different versions were used for the “pre” and “post” tests (forms A and B), and the order of the forms was counterbalanced between participants.

##### Expressive Vocabulary Test-Third Edition (Expressive Vocabulary Test-3)

This standardized test (Williams, 2018) is designed to assess expressive vocabulary and word retrieval based on words in Standard American English in children and adults. Different versions were used for the “pre” and “post” tests (forms A and B), and the order of the forms was counterbalanced between participants.

##### Phonological Awareness Literacy Screening Quick Checks for K-3rd Grades Test (PALS-Quick Checks)

This standardized test (Invernizzi et al., 2003) is designed to assess early literacy skills, specifically phonological and print awareness in preschoolers. We administered the pseudoword decoding (set A) sub-part of this test. Different versions were used for the “pre” and “post” tests (forms 1 and 2), and the order of the forms was counterbalanced between participants.

All the described tests were adapted to online administration by uploading the stimuli to PowerPoint presentations and were presented to the participants on the screen during the “pre” and “post” online sessions *via* Zoom. For the WRMT-III RAN subtest, the participants got a hard copy of the stimuli sheet and were instructed to use it during the online sessions.

### Data Analysis

First, the experimental and the control groups were compared on their baseline scores for each test, incorporating a two-sample *t*-test to ensure that the two groups did not differ significantly in their baseline “pre” scores. Since the pseudoword decoding scores are not normally distributed, we incorporated a Mann-Whitney U Test to compare between groups for this measure. The Mann-Whitney U Test is a non-parametric test suitable for independent samples. Second, to measure the effectiveness of the training program, statistical analysis incorporated separate repeated-measures ANOVA for each test with raw scores as dependent variables, session (“pre” vs. “post”) as within-subject factors, and group (experimental vs. control) as a between-subject factor. Further, we planned *post hoc* paired-sample *T*-tests to examine the “pre” and “post” results per group. Since the pseudoword decoding scores are not normally distributed, we incorporated a Wilcoxon Signed-Rank Test to compare “pre” and “post” results for this measure within each group. The Wilcoxon Signed-Rank Test is a non-parametric test suitable for dependent samples. Finally, to measure the potential effect of SES, we measured the correlations between the baseline performance across groups and the SES measures of parental education and income-to-need ratio. Finally, we added the SES measures together with the repeated-measures AVOVA group comparison to examine whether the results are affected by SES factors.

## Results

First, as shown in Table 3, the experimental and the control groups did not differ significantly on the baseline scores for the standardized and non-standardized tests. Second, the repeated measures ANOVA showed a significant session-by-group interaction for the specific skills that were taught in the online Reading Camp: phonological awareness, *F*(1,113) = 26.664, *p* < 0.001, eta squared = 0.191; lowercase letters’ names, *F*(1,114) = 7.328, *p* < 0.01, eta squared = 0.06, and sounds, *F*(1,114) = 10.672, *p* < 0.01, eta squared = 0.086. Interestingly, the repeated measures ANOVA also revealed a significant session-by-group interaction for uppercase letters’ sounds, *F*(1,113) = 4.591, *p* < 0.05, eta squared = 0.039, and for pseudoword decoding, *F*(1,113) = 4.3, *p* < 0.05, eta squared = 0.037, despite not being directly taught in the online Reading Camp.

**TABLE 3:** Comparisons between the experimental and control group on the baseline measurements.

Test	Experimental Group		Control Group		Comparison			
	Mean	*SD*	Mean	*SD*	Test	Value	*df*	sig
Uppercase letters’ names	19.34	7.53	18.36	7.86	*T*-test	0.620	114	0.536
Uppercase letters’ sounds	7.57	6.09	5.45	5.09	*T*-test	1.901	69.96	0.061
Lowercase letters’ names	15.60	6.87	15.73	6.77	*T*-test	–0.089	114	0.929
Lowercase letters’ sounds	6.35	5.73	5.15	4.69	*T*-test	1.065	114	0.289
Phonological Awareness (WRMT-III)	14.22	5.86	14.06	5.202	*T*-test	0.134	114	0.894
Rapid Automatic Naming (WRMT-III)	13.10	3.84	13.73	3.83	*T*-test	–0.714	94	0.477
Pseudoword decoding (PALS-Quick Checks)	1.10	3.05	0.91	2.403	Mann-Whitney U Test	–1356	114	0.900
Expressive vocabulary Test-3rd edition	82.29	13.03	81.97	12.95	*T*-test	0.119	114	0.905

*All results are calculated for the raw scores for each test. For the Uppercase letters’ sounds, the Levene’s test for equality of variances was significant. Hence, we present the results relevant for unequal variances.*

**FIGURE 3:**
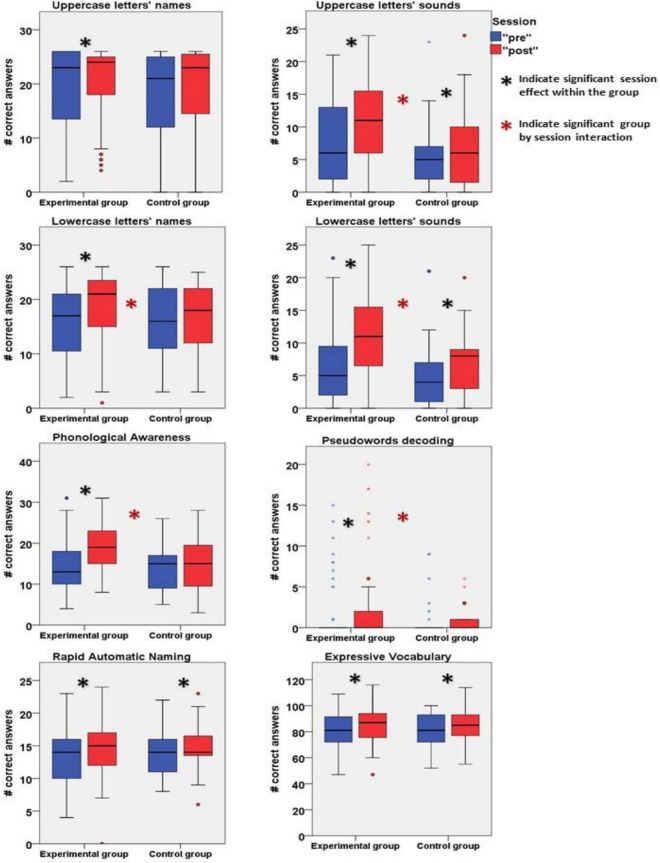
Experimental and control groups’ results of “pre” and “post” measurements.

We further examined the planned *post hoc* paired-sample *T*-test for the “pre” and “post” tests per group. As shown in Table 4, we found that the experimental group significantly improved on all the standardized and non-standardized tests. However, the control group only improved on uppercase letters’ sounds, lowercase letters’ sounds, rapid automatic naming, and expressive vocabulary. The group comparison results are demonstrated in Figure 3. Furthermore, as shown in Table 5, the correlations between parental education and the baseline performance across groups were non-significant for all baseline measures. However, the correlations between the income-to-need ratio and the Phonological Awareness and Pseudoword Decoding baseline scores were significant. In contrast, all other scores were not significantly correlated with the income-to-need ratio. Finally, when controlled for the SES measures together in the repeated-measures ANOVA model with group comparison, the session-by-group interaction remained significant for uppercase letters’ sounds, *F*(1,89) = 4.419, *p* < 0.05, eta squared = 0.047, lowercase letters’ names, *F*(1,90) = 7.311, *p* < 0.01, eta squared = 0.075, and sounds, *F*(1,90) = 12.592, *p* < 0.01, eta squared = 0.123, and phonological awareness, *F*(1,89) = 29.604, *p* < 0.001, eta squared = 0.250. However, it was no longer significant for pseudowords decoding, *F*(1,89) = 3.502, *p* = 0.065, eta squared = 0.038.

**TABLE 4:** Paired-sample *T*-test for the “pre” and “post” measures by group.

Test	Group	“pre”		“post”		Comparison			
		
		Mean	*SD*	Mean	*SD*	Test	Value	*df*	sig
Uppercase letters’ names	Experimental	19.34	7.53	20.28	6.56	*T*-test	4.11	82	< 0.001
	Control	18.94	7.25	19.59	7.68	*T*-test	1.18	31	0.244
Uppercase letters’ sounds	Experimental	7.57	6.09	10.92	6.24	*T*-test	6.71	82	< 0.001
	Control	5.63	5.07	7.03	5.80	*T*-test	2.08	31	0.045*
Lowercase letters’ names	Experimental	15.60	6.87	18.72	6.26	*T*-test	6.39	82	< 0.001
	Control	15.73	6.77	16.67	6.66	*T*-test	1.82	32	0.077
Lowercase letters’ sounds	Experimental	6.35	5.73	11.12	6.20	*T*-test	9.40	82	< 0.001
	Control	5.15	4.69	7.09	4.97	*T*-test	3.83	32	0.001**
Phonological Awareness (WRMT-III)	Experimental	14.22	5.86	18.80	5.49	*T*-test	10.16	82	< 0.001
	Control	14.34	5.02	14.66	6.31	*T*-test	0.492	31	0.604
Rapid Automatic Naming (WRMT-III)	Experimental	13.28	3.80	15.00	3.75	*T*-test	4.77	60	< 0.001
	Control	13.83	3.95	14.88	3.89	*T*-test	1.89	23	0.042*
Pseudoword decoding (PALS-Quick Checks)	Experimental	1.10	3.05	2.14	4.48	Wilcoxon Signed-Rank Test	–3.507	82	< 0.001
	Control	0.94	2.40	0.75	1.54	Wilcoxon Signed-Rank Test	–0.206	31	0.837
Expressive vocabulary Test-3rd edition	Experimental	82.29	13.03	85.46	13.02	*T*-test	3.48	82	0.001**
	Control	81.97	12.95	85.79	12.58	*T*-test	2.56	32	0.015*

*All results are calculated for the raw scores for each test.*

*Significance level <0.05.

**Significance level <0.01.

**TABLE 5:** Correlation between SES measures and the baseline performances across groups.

Test		Income-to-needs ratio			Parental education (Average number of years)		
	Correlation test	Value	Significant	*N*	Value	Significant	*N*
Uppercase letters’ names	Pearson	0.054	0.613	90	–0.035	0.708	116
Uppercase letters’ sounds	Pearson	0.054	0.615	90	–0.016	0.865	116
Lowercase letters’ names	Pearson	0.188	0.075	90	–0.046	0.625	116
Lowercase letters’ sounds	Pearson	0.125	0.241	90	–0.040	0.669	116
Phonological Awareness (WRMT-III)	Pearson	0.316	0.002**	90	0.076	0.418	116
Rapid Automatic Naming (WRMT-III)	Pearson	0.134	0.247	76	0.021	0.843	96
Pseudoword decoding (PALS-Quick Checks)	Spearman	0.221	0.036*	90	0.022	0.814	116
Expressive vocabulary Test-3rd edition	Pearson	0.182	0.085	90	0.173	0.063	116

*All results are calculated for the raw scores for each test.*

*Significance level <0.05.

**Significance level <0.01.

## Discussion

The first goal of the current research was to examine whether pre-K children can learn fundamental reading skills in an online environment. Despite the challenges and potential drawbacks of online intervention programs, it is valuable to test the efficacy of translating in-person interventions to online programs. Online intervention programs have the potential to scale up without exhausting resources and increase access to diverse populations (Gijbels et al., 2021; Ozernov-Palchik et al., 2022). Furthermore, amid the COVID-19 pandemic, there is an urgent need to examine the crucial components of early reading acquisition and how it can be taught in an online environment in times of school closure and more generally when online instruction could potentially reach children who cannot attend school. In the current study, we developed the online Reading Camp based on its in-person equivalent (Yeatman et al., 2022), to test the efficacy of an online early reading program. The online Reading Camp program focused on fundamental skills that promote reading acquisition (phonological awareness and letter-sound knowledge) by incorporating evidence-based systematic instructions and engaging activities. We examined the effectiveness of the online Reading Camp by comparing the results between children who participated in this 2-week program and no-treatment control participants. We further examined whether 5-year-old pre-K children’s ability to benefit from the online Reading Camp is related to their SES. The second goal was to set the ground for future examination of the structural and functional changes that occur in the brain when children learn to read.

Our results indicate that 5-year-old pre-K children can benefit from a well-structured evidence-based reading instruction program, even if online and short-term. Children who participated in the online Reading Camp showed significant improvements compared to no-treatment control participants on skills directly taught in the program, namely, phonological awareness and lowercase letters’ names and sounds, as measured by standardized and non-standardized tests. Interestingly, despite not directly being taught during the online Reading Camp, the experimental group also showed significant improvements compared to the control group on uppercase letters’ sounds and pseudowords decoding. These results indicate that the online Reading Camp participants could generalize what they have learned to other reading-related skills. Notably, both experimental and control group participants improved on uppercase and lowercase letters’ sounds. However, the effect was more prominent for the experimental group as reflected by the significant group-by-session interaction and the significance level of the paired sample T-tests. Furthermore, despite not showing a significant group-by-session interaction, only the online Reading Camp participants showed improvement in uppercase letters’ names.

Our results replicate and extend the results from the in-person LLC (Yeatman et al., 2022) and support the conclusions of the NRP report (National Reading Panel [NRP], 2020) that phonological awareness and letter-sound knowledge instructions effectively teach young children the foundation of reading, especially when incorporating systematic and explicit instructions that combine both components in engaging activities and are delivered in small groups. The current results expand the findings from the in-person LLC and the NRP report by indicating, for the first time, that programs that include all these qualities are effective for 5-year-old pre-K children even when administered entirely online over a 2-week period. While previous studies showed the benefit of computer-based programs in teaching young children phonological awareness and letter-sound knowledge (Mitchell and Fox, 2001; Lonigan et al., 2003; Segers and Verhoeven, 2005; Macaruso and Walker, 2008; de Graaff et al., 2009; Wild, 2009; Volpe et al., 2011; Savage et al., 2013; Prescott et al., 2018), the current study is the first to demonstrate the effectiveness of a fully online teacher-led program for this age range.

The results of the current study further show that while children’s phonological awareness and pseudoword decoding pre-camp skills were positively correlated with the income-to-need ratio across groups, SES measures did not affect the results for measures other than the pseudowords decoding. This result provides evidence that the online Reading Camp program is effective regardless of the child’s SES level. These results are in line with previous studies that demonstrated correlations between SES measures and phonological awareness skills (Bowey, 1995; Lonigan et al., 1998; Locke et al., 2002; McDowell et al., 2007; Lundberg et al., 2012), as well as with meta-analysis that showed the effectiveness of phonological awareness and letter-sound knowledge instructions across SES (Ehri et al., 2001; National Reading Panel [NRP], 2020). However, further research is needed to examine whether the long-term effects of early instruction are robust and durable across SES levels.

Notably, both experimental and control group participants improved on the Expressive Vocabulary Test (EVT) and Rapid Automatic Naming (RAN) measures. Since the skills measured by these tests were not the focus of the online Reading Camp program and we did not expect to find improvement in these measures, the improvement could reflect a test-retest practice effect. The test-retest practice effect is a widely documented phenomenon attributed to different factors such as familiarity with the test setting and material and memory, especially when the tests are completed within a relatively short time window (Hausknecht et al., 2007). The exact form was used for the “pre” and “post” for the RAN test. However, different versions were used for the EVT. While test-retest is more pronounced when using identical forms, it has been found for different versions (Hausknecht et al., 2007). The improvement demonstrated on the RAN and EVT measures across groups highlights the importance of including a control group when measuring the effectiveness of any intervention or training program. The comparison between the experimental and control group results highlights the effectiveness of the online Reading Camp by demonstrating significant differences between the groups on measures that are specific (phonological awareness and letter-sound knowledge) or closely related to (pseudowords decoding) the content taught in the online Reading Camp.

### Limitations and Future Directions

An important issue to consider is the characteristics of the control group in this study. Although the experimental and control groups did not differ on the demographic measures and the baseline scores of the standardized and non-standardized tests, they differed in size. The experimental group included 83 participants, and the control group included 33 participants. Furthermore, the experimental group interacted with the teachers and other participants during the 2-week online Reading Camp, while the control group did not interact with the teachers and other participants between the “pre” and “post” sessions. Future studies could consider including comparably-sized group and a control condition in which control participants participate in an alternative camp program, strengthening the conclusion regarding the effectiveness of the specific online Reading Camp program. We note that the results of the in-person RTC design, which compared the effectiveness of two different intervention programs, suggest that it is the specific content of the online and in-person Reading Camp programs that produce the results (Yeatman et al., 2022). In addition, as a group, the control participants were slightly older than the experimental group. Half of the control participants (and only one experimental group participant) had begun to attend kindergarten after enrolling in the current 2-week study. Control participants’ gains in the skills measured in the “post” sessions may be attributed to this early entry into school. In the future, online Reading Camp studies should include only experimental and control participants that have not begun kindergarten during the 2-week study.

With the potential to scale up this online Reading Camp program, the second goal of the current study was to set the ground for future examination of how children’s first experiences with reading tune the underlying structure and function of the brain’s visual and language pathways to enable reading. The evidence presented here, alongside previous evidence for the efficacy of the in-person program, indicates that the online Reading Camp program is effective and can be used to tackle a variety of questions regarding structural and functional plasticity in the early stages of reading acquisition.

Reading is a recent evolutionary milestone in human history. As such, it must emerge from brain networks that evolved for other purposes (such as language and visual processing), following explicit instructions and practice (Yeatman and White, 2021). Studies that examined the developmental trajectory of the neural networks related to reading found the emergence of specialization to printed words within a specific region in the left fusiform gyrus termed “the visual word form area” and its connectivity with brain regions related to language and visual processing (Yamada et al., 2011; Wang et al., 2017; Centanni et al., 2018; Dehaene-Lambertz et al., 2018; Yu et al., 2018). However, the amount of instruction and practice needed for these changes to emerge is unknown. Moreover, we do not know how children’s first experiences with reading tune the language and visual brain networks. A recent Magnetoencephalography (MEG) study highlighted the rapid plasticity that occurs as children begin learning to read in the in-person Reading Camp program (Yeatman et al., 2022), and there is a wealth of important questions that can be examined, related to the developmental trajectory in brain networks related to reading acquisition. Our next project will follow up on these MEG findings and incorporate MRI and fMRI to examine whether our 2-week online Reading Camp can induce the emergence of these brain changes.

Another goal is to examine the long-term outcomes of this intensive, short-term, online Reading Camp program on behaviorally measured reading skills as well as the reading brain network. We plan to follow up longitudinally with the current online Reading Camp participants to measure these long-term effects on later reading outcomes and brain-related changes.

Finally, we plan to directly compare the in-person to the online Reading Camp programs to address the efficacy of the online program and whether it yields comparable outcomes as an in-person program.

## Data Availability Statement

The raw data supporting the conclusions of this article will be made available by the authors, without undue reservation.

## Ethics Statement

The studies involving human participants were reviewed and approved by University of Washington Human Subjects Division IRB Committee D. Written informed consent from the participants’ legal guardian/next of kin was not required to participate in this study. Written informed consent was not obtained from the individual(s), nor the minor(s)’ legal guardian/next of kin, for the publication of any potentially identifiable images or data included in this article.

## Author Contributions

YW, PK, and JY contributed to the conception and design of the study. JY, LG, and SE designed the in-person intervention programs. YW, LG, SE, HL, JM, and BW adapted the in-person intervention program to an online program. YW organized the database, performed the statistical analysis, and wrote the first draft of the manuscript. YW, PK, and JY contributed to manuscript revision, read, and approved the submitted version.

## Conflict of Interest

The authors declare that the research was conducted in the absence of any commercial or financial relationships that could be construed as a potential conflict of interest.

## Publisher’s Note

All claims expressed in this article are solely those of the authors and do not necessarily represent those of their affiliated organizations, or those of the publisher, the editors and the reviewers. Any product that may be evaluated in this article, or claim that may be made by its manufacturer, is not guaranteed or endorsed by the publisher.

## Footnotes

1 Participants’ parents gave informed consent for all study procedures both verbally and by approving the electronic consent form, which they read (via REDCap). The UW IRB committee waived the requirement of written informed consent for all study procedures including participation in the online reading camp and the publication of identifiable images.
